# Protocol of a randomized trial of acceptance and commitment therapy for patient fatigue interference and caregiver burden in advanced gastrointestinal cancer

**DOI:** 10.1016/j.cct.2025.108168

**Published:** 2025-11-26

**Authors:** Catherine E. Mosher, Eileen H. Shinn, Elizabeth L. Addington, Wei Wu, Jonathan B. Bricker, Paul R. Helft, Anita A. Turk, Laura B. Vater, Ashiq Masood, Shadia I. Jalal, Patrick J. Loehrer, Victoria L. Champion, Shelley A. Johns

**Affiliations:** a Department of Psychology, Indiana University Indianapolis, 402 North Blackford Street, LD 124, Indianapolis, IN 46202, USA; b Department of Behavioral Science, The University of Texas M.D. Anderson Cancer Center, 1515 Holcombe Blvd, Unit 1330, Houston, TX 77030, USA; c Department of Medical Social Sciences, Northwestern Feinberg School of Medicine, 625 N. Michigan Avenue, 17^th^ Floor, Chicago, IL 60611, USA; d Division of Public Health Sciences, Fred Hutchinson Cancer Research Center, 1100 Fairview Avenue North, PO Box 19024, M3-B232, Seattle, WA 98109, USA; e Department of Psychology, University of Washington, 119 Guthrie Hall, Seattle, WA 98195, USA; f Indiana University Melvin and Bren Simon Comprehensive Cancer Center, Indiana Cancer Pavilion, 535 Barnhill Drive, Suite 473, Indianapolis, IN 46202, USA; g Indiana University School of Nursing, 1111 Middle Drive, NU 340G, Indianapolis, IN 46202, USA; h Indiana University School of Medicine, Center for Health Services Research, Regenstrief Institute, 1101 W. 10^th^ Street, Indianapolis, IN 46202, USA

**Keywords:** Acceptance and commitment therapy, Advanced gastrointestinal cancer, Caregiving burden, Fatigue, Psychosocial intervention, Quality of life

## Abstract

Fatigue’s interference with activities, mood, and cognition is one of the most prevalent and distressing problems of patients with advanced gastrointestinal cancer. As fatigue interferes with patient functioning, family caregivers often report feeling burdened by increasing demands. Evidence-based interventions for patient fatigue interference and caregiver burden are lacking in advanced gastrointestinal cancer. In a pilot trial, telephone-based Acceptance and Commitment Therapy (ACT) showed potential for reducing patient fatigue interference and caregiver burden in this population. The current Phase II trial seeks to determine the efficacy of this intervention for patients with advanced gastrointestinal cancer and moderate-to-severe fatigue interference and their family caregivers with significant caregiving burden. In this trial, 244 dyads are randomly assigned to either the ACT intervention or an education/support control. Participants in both conditions attend six weekly 50-min telephone sessions, four of which involve both dyad members, and a 30-min booster session. The primary aim is to test the effects of telephone-delivered ACT on patient fatigue interference and caregiver burden. Secondary outcomes include patient sleep interference and patient and caregiver engagement in daily activities and quality of life. Outcomes are assessed at baseline, 2 weeks post-intervention, and 3 months post-intervention. This trial also examines whether increased psychological flexibility, defined as mindful acceptance of present experiences, including challenges, while pursuing actions aligned with personal values, mediates ACT’s effects on primary outcomes. Our ability to demonstrate ACT’s efficacy will support its adoption in cancer care. Findings will also inform future ACT trials for dyads coping with other serious illnesses.

## 1. Introduction

Fatigue is a prevalent, disabling symptom in patients with advanced gastrointestinal (GI) cancer [1–4]. Moderate-to-severe fatigue is reported by up to 68 % of patients with metastatic colorectal cancer [1,2]. Among patients with GI cancer, fatigue has been associated with impaired daily activities and other symptoms [1,5–8]. As symptoms interfere with patient functioning, family caregivers face increased demands that affect their quality of life (QoL) [9–11]. Among caregivers of patients with colorectal cancer, greater caregiving burden, defined as the impact of caregiving on various aspects of their lives, has been associated with worse QoL [12]. As colorectal cancer incidence increases among younger adults [13], their caregivers often experience significant stressors, including balancing multiple roles and heightened economic strain [14,15].

Evidence-based interventions for addressing patient fatigue and caregiver burden in advanced cancer are lacking. Meta-analyses of pharmacologic and behavioral interventions for fatigue in advanced cancer characterized the evidence as inconclusive and highlighted the small samples in many studies [16,17]. Additionally, behavioral interventions for cancer caregivers have produced small effects on caregiving burden and QoL [18,19]. Meta-analytic findings suggest that psychotherapy and skills training interventions show greater promise for decreasing cancer caregiver burden than psycho-education/support interventions [19]. Most of these intervention trials did not focus on caregivers of late-stage patients and did not have a distress criterion for eligibility. Thus, effect sizes may have been larger if the trials had focused on subgroups most in need of intervention.

One behavioral intervention with strong potential for reducing cancer patient and caregiver suffering is acceptance and commitment therapy (ACT) [20,21]. Rather than aiming to reduce symptoms, the goal of ACT is to increase psychological flexibility so that unwanted internal experiences (e.g., symptoms, feelings, thoughts) interfere less with valued activities [22,23]. Psychological flexibility is a multidimensional construct comprised of mindfulness/acceptance processes (i. e., nonjudgmental attention to the present moment) and commitment/behavior change processes (i.e., identifying personal values and taking action steps aligned with these values) [22,23]. ACT has shown evidence of feasibility and acceptability in pilot trials with patients and patient-caregiver dyads coping with advanced cancer [21,24–26]. In our pilot RCT, 40 advanced GI cancer patient-caregiver dyads were randomized to six telephone sessions of either ACT or time-equivalent education/support [21]. ACT showed strong evidence of feasibility and promise in reducing patient fatigue interference with functioning and caregiver burden. Feasibility was demonstrated by the eligibility screening rate (54 % of reached patients and 96 % of reached, patient-identified caregivers agreed to be screened), consent rate (100 % of eligible patients and caregivers consented), and retention (81 % of participants at 2 weeks post-intervention and 73 % at 3 months post-intervention). Most attrition (82 %) was due to severe medical problems or death.

The primary aim of this NIH-funded Phase II RCT is to examine the efficacy of our telephone-delivered ACT intervention for patients with advanced GI cancer and fatigue interference and their family caregivers with caregiver burden. We hypothesize that ACT will lead to improved primary outcomes of patient fatigue interference and caregiver burden compared to time-matched education/support. Our second aim is to examine ACT’s effects on the secondary outcomes of patient sleep interference and patient and caregiver engagement in daily activities and QoL. We hypothesize that ACT will lead to improved secondary outcomes compared to education/support. Our third aim is to test the hypothesis that increased psychological flexibility will mediate ACT’s effects on patient fatigue interference and caregiver burden. Our fourth aim is to explore changes in the two core aspects of psychological flexibility (i.e., mindfulness/acceptance and commitment/behavior change processes) as mediators of ACT’s effects on patient fatigue interference and caregiver burden. This paper describes the rationale, study design, methods, and statistical plan.

## 2. Materials and methods

### 2.1. Overview of study design

This Phase II RCT tests the effects of telephone-based ACT on patient fatigue interference and caregiver burden in advanced GI cancer. We plan to enroll 244 patient-caregiver dyads over about four years. Dyads are randomized to six weekly 50-min telephone sessions of ACT or six weekly 50-min telephone sessions of education/support. Outcomes are assessed at baseline, 2 weeks post-intervention, and 3 months post-intervention (primary endpoint). Dyads in both conditions also complete a 30-min booster phone session one month after the 2-week follow-up. This trial is registered in clinicaltrials.gov (NCT06532877) and approved by the Indiana University Institutional Review Board.

### 2.2. Participant eligibility criteria

To enroll in this study, both the patient and caregiver need to be eligible and consent to participate. Patient eligibility criteria are as follows: a) unresectable stage III or IV GI cancer according to medical records and the oncologist, b) no significant cognitive impairment as ascertained by medical records and a mental status questionnaire (<3 errors) [27], c) in bed or chair less than half the day (patient-reported Eastern Cooperative Oncology Group [ECOG] score ≤ 2) [28], d) not enrolled in hospice, e) has an eligible, consenting family caregiver, and f) elevated fatigue interference (mean score ≥ 2.5 on the Fatigue Interference subscale of the Fatigue Symptom Inventory [FSI]) [29,30]. In our pilot research in advanced cancer, this level of fatigue interference was significantly associated with clinically meaningful fatigue (score ≥ 3 on the FSI composite measure) [31]. Caregiver eligibility criteria are as follows: a) family or friend caregiver who either lives with the patient or has visited them at least twice a week for the past month, and b) significant caregiving burden (score ≥ 4 on the 4-item Zarit Burden Interview, score ≥ 6 on the 6-item Zarit Burden Interview, or score ≥ 7 on the 7-item Zarit Burden Interview [32]). Clinical cutoffs on the caregiving burden measures have been established in advanced cancer caregivers [32]. Other patient and caregiver eligibility criteria are: a) ≥18 years of age, b) English fluency, c) working phone service, and d) no hearing impairment that precludes study participation.

### 2.3. Recruitment

Potentially eligible patients are identified via medical record review and consultation with the oncologist. Patients receive recruitment materials and a consent form via the hospital’s email system or postal mail. Materials include contact information for those who have questions or wish to opt out.

Study staff call patients who do not opt out at least 3 days after emailing the recruitment materials or about 1 to 2 weeks after mailing the materials. The staff member describes the study as testing telephone support programs for stress and symptoms and answers questions. Then the staff member administers a screening assessment (i.e., identification of caregiver, Fatigue Interference subscale of FSI [29,30], ECOG [28], and mental status questionnaire [27]) with the patient’s permission. Eligible and interested patients provide verbal consent for study participation over the phone. Caregivers of consenting patients receive recruitment materials via email or postal mail followed by telephone calls for eligibility screening and consent. Other patients and caregivers are approached during an oncology clinic visit for eligibility screening and written informed consent.

### 2.4. Randomization and blinding

Following baseline assessments, dyads are randomly assigned to ACT or education/support using stratified block randomization to balance the groups by patient age (<65 yrs. vs. ≥65 yrs.) and performance status (patient-reported ECOG scores 0 or 1 vs. 2 [28]). Performance status informs treatment decisions for advanced GI cancer [33]. The statistician uses the R package “blockrand” [34] to generate random assignments within blocks. Randomly varying block sizes of 2 and 4 are used to maintain allocation concealment and ensure balanced allocation to study conditions. Following randomization, study condition assignments are identifiable to participants, interventionists, their supervisors, and staff mailing intervention materials. Other investigators, outcome assessors, and data analysts are blinded.

### 2.5. Interventions

#### 2.5.1. Acceptance and commitment therapy (ACT)

**Fig. 1.**
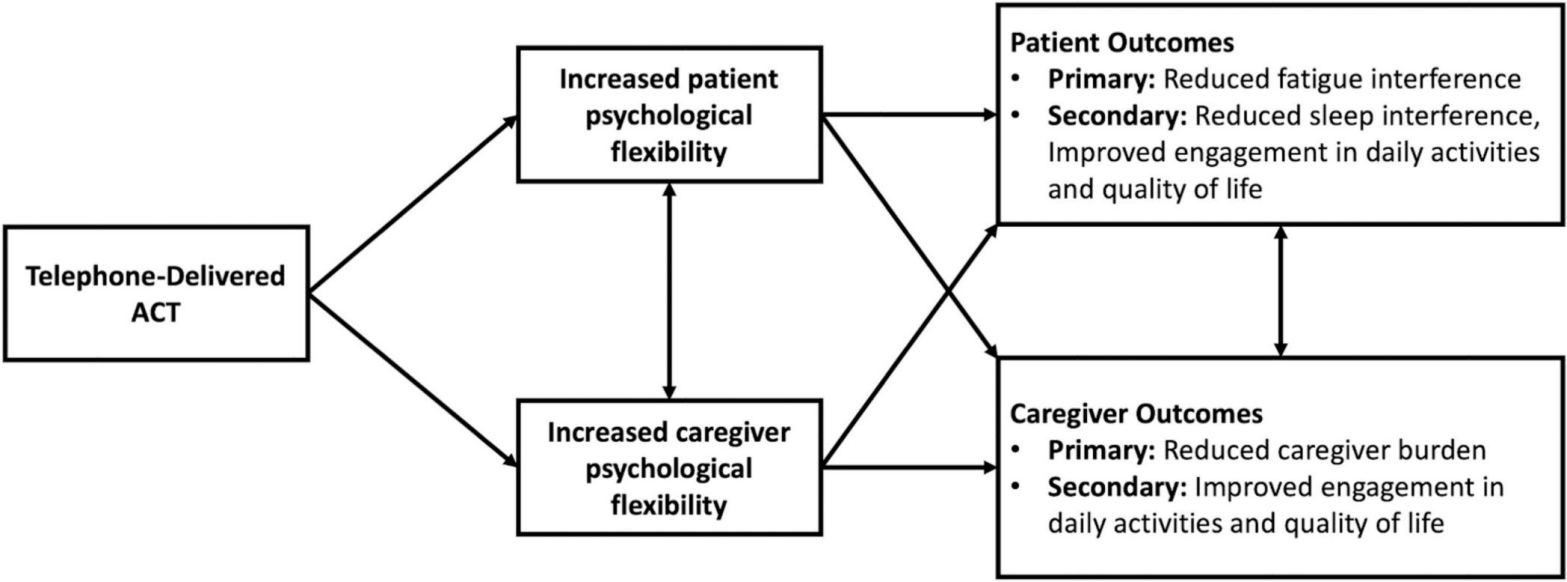
Conceptual model.

Study team members developed the ACT manual, which was informed by literature on the experiences of patients with advanced GI cancer and caregivers [1–3,10,35–37], the ACT model [20,22], prior ACT trials with cancer and other medical patients [21,24–26,38], and our clinical experience. Our team tested components of the intervention (e.g., mindfulness, values-based action) in our ACT trial targeting fatigue interference in patients with metastatic breast cancer [26]. Intervention components are summarized in Table 1. Based on the ACT model [20,22], the intervention addresses patient fatigue interference and caregiver burden through increasing psychological flexibility (see Fig. 1: Conceptual Model). Psychological flexibility is expected to increase through practicing mindfulness (e.g., meditations that encourage compassionate awareness of current internal experiences), learning adaptive coping strategies (e.g., acceptance, perspective-taking), identifying personal values (e.g., staying connected with family), and engaging in values-based actions. Participants set values-based action goals in the SMART format (Specific, Measurable, Achievable, Relevant, and Time-Bound).

Patients and caregivers jointly complete sessions 1 and 4–6 via speakerphone, whereas sessions 2 and 3 are separately delivered to patients and caregivers. We adapted ACT to the dyad by including joint mindfulness practices and a focus on their relationship during discussions. The therapist supports both participants in practicing compassionate acceptance of their own and each other’s thoughts, mitigating defensive reactions and fostering open communication. Individual sessions with patients and caregivers include discussing personal values. Additionally, the utility of attempts to avoid fatigue (for patients) or difficult thoughts and feelings about caregiving (for caregivers) is discussed. The emphasis in individual sessions is shifting from avoidance to pursuing values-based actions despite experiencing fatigue or caregiving burden.

During each session, patients complete three FSI items (i.e., average fatigue severity and fatigue interference with general activity and enjoyment of life) [29,30], and both patients and caregivers complete two Patient-Reported Outcomes Measurement Information System (PROMIS) depression items and two PROMIS anxiety items [39]. These assessments allow the therapist to monitor symptoms and may be examined in exploratory analyses. Each person’s home practice of ACT skills is also documented, and practice for the week ahead is discussed. One month after the 2-week follow-up, the same therapist holds a 30-min call with the dyad to reinforce ACT-based skills. Each person is sent handouts summarizing session topics and audio recordings that our team developed to guide mindfulness practices.

#### 2.5.2. Education/support

The education/support condition was tested in our pilot trial [21] and is comparable to controls in other cancer trials [40,41]. Dyads in this condition discuss their fatigue and other cancer-related concerns with a therapist providing reflective listening and empathic responses. The therapist directs dyads to resources for practical and health information and mental health services. The length of sessions and number and order of dyadic vs. individual sessions are the same as those for the ACT condition. Additionally, the same fatigue, depression, and anxiety assessments as those in the ACT condition are administered during each session [29,30,39]. Table 1 provides an overview of the education/support components. Sessions include an orientation to the patient’s hospital and treatment team, education about prevalent QoL concerns and symptoms experienced by patients with cancer and caregivers, and referrals to medical center and community resources for addressing these concerns. Therapists also describe resources for addressing financial concerns and methods of evaluating health information. One month after the 2-week follow-up, the same therapist holds a 30-min call with the dyad to reinforce concepts and encourage use of resources as needed. Each person receives handouts summarizing session topics and is asked to review them as homework. ACT concepts are not discussed.

#### 2.5.3. Training and supervision of therapists

Therapists are psychologists, social workers, certified life coaches through an International Coach Federation (ICF)-accredited program, or advanced doctoral students in clinical psychology with extensive training and experience delivering ACT or supportive counseling to medical populations. Each intervention is delivered by different therapists, and both conditions include therapists at diverse educational and career stages. Therapists received initial education in advanced GI cancer diagnosis and treatment, psychological distress, and either ACT or supportive counseling techniques. Initial training also included roleplays of sessions detailed in manuals. All sessions are digitally recorded, and fidelity monitors randomly select recordings to review for adherence to the manual using checklists developed in our pilot research. Additionally, seven items adapted from prior research [42–44] assess how consistently ACT therapists’ behaviors align with the ACT model. Individuals monitoring adherence have expertise in ACT or supportive counseling and were trained in fidelity monitoring. During weekly or biweekly supervision with therapists, clinical psychologists provide treatment adherence scores (number of required topics covered in each session/total number of fidelity criteria), and treatment fidelity and quality issues are discussed. Supervisor feedback is implemented in subsequent sessions.

### 2.6. Retention

Several strategies are used to increase study retention. All study personnel have been trained to clearly communicate study expectations to participants and to offer the option of completing follow-ups if they do not adhere to the intervention. Use of the telephone for all assessments and the intervention has resulted in strong retention in prior trials [21,45,46] and reduces barriers to participation for individuals with high symptom burden or low income. Text, phone, or email reminders are sent to participants before appointments. Each participant receives $40 in gift cards for participating in each of the three assessments, for a possible total of $120 in gift cards.

### 2.7. Study measures and data collection schedule

Study measures and the data collection schedule are shown in Table 2. REDCap is used to store study data. Patients and caregivers individually complete a 35-min baseline assessment and two, 30-min follow-up assessments conducted by blinded staff. All outcomes and mediators have evidence of internal consistency reliability and construct validity in cancer, general population, or caregiving samples [3,29,30,32,47–56].

#### 2.7.1. Primary outcomes

The primary outcome measure for patients is the 7-item Fatigue Interference subscale of the FSI [29,30]. Items evaluate the extent to which fatigue in the past week interfered with activities, mood, and cognition on 11-point scales (0 = no interference; 10 = extreme interference). The primary outcome measure for caregivers is the 12-item Zarit Burden Interview [32,47], which assesses personal strain and role strain due to caregiving on 5-point scales (0 = never; 4 = nearly always).

#### 2.7.2. Secondary outcomes

Patient *sleep interference* is assessed with the 8-item PROMIS sleep-related impairment measure, which evaluates the perceived interference of sleep problems with functioning [48,49]. Patient and caregiver e*ngagement in daily activities* is assessed with the 6-item PROMIS short-form measure of ability to participate in social roles and activities [50]. The items, which are reverse-coded, evaluate difficulty participating in social and recreational activities as well as usual work (including housework). Patient and caregiver *QoL* is measured with the 10-item PROMIS measure of global health, including physical, mental, and social well-being [51].

#### 2.7.3. Mediators

Patients and caregivers complete three measures of potential mediators. *Psychological flexibility,* as measured by the 7-item Acceptance and Action Questionnaire-II (AAQ-II) [52], is assessed as a hypothesized mediator of ACT’s effect on primary outcomes. The two core components of psychological flexibility are also evaluated as exploratory mediators. First, *mindfulness/acceptance* is assessed with the 10-item Cognitive and Affective Mindfulness Scale-Revised (CAMS-R) [53]. Items measure attention, present-focus, awareness, and acceptance/non-judgement. Second, *commitment/behavior change* is assessed with the 5-item Value Progress subscale of the Valuing Questionnaire [54]. This measure evaluates progress in living according to personal values.

#### 2.7.4. Descriptive variables

Demographic and medical variables are evaluated to characterize the sample and to assess equivalence across study conditions. Caregivers also report their relationship to the patient and whether they live with the patient. Medical comorbidities are assessed at baseline only; all other medical factors are collected at each timepoint. Patients and caregivers complete a checklist of eight or nine chronic medical conditions, respectively [57]. Patient functional status is also measured with a 1-item self-reported ECOG measure [28]. Patient cancer information is collected via chart review. Patients and caregivers also report their physical and mental healthcare use in five domains (e.g., number of emergency room visits, outpatient visits) in the past 3 months at baseline and over the study period (past 2 months and 3 months at first and second follow-ups, respectively) [57,58]. Additionally, participants report prescribed and over-the-counter medications [59,60].

Finally, measures of symptom severity are administered at all timepoints to characterize the sample. For patients and caregivers, fatigue severity is measured with four FSI items [29,30], and sleep disturbance, cognitive concerns, anxiety, and depressive symptoms are each assessed with a 4-item PROMIS measure [39,48,49,61–63]. For patients, incontinence and diarrhea are assessed with 4-item and 6-item PROMIS measures, respectively [64].

## 3. Statistical analyses

### 3.1. Preliminary analyses

#### 3.1.1. Missing data/attrition

We will examine demographic and medical factors at baseline that might predict dropout using logistic regression. For Aims 1 and 2 described below, all randomized participants will be included in intent-to-treat analyses [65]. Second, for participants who miss assessments, we will use full information maximum likelihood (FIML) to handle missing data without disregarding any incomplete cases.

#### 3.1.2. Analyses of potential covariates

*T*-tests and chi-square analyses will be used to examine differences between study conditions on potential baseline covariates (e.g., demographics, medical factors). Any differences will be considered when interpreting results, and factors that differ between groups will be included as covariates in sensitivity analyses.

### 3.2. Analysis for aim 1

Linear mixed effects modeling (R lme4) [66] will be used to test the hypothesis that ACT will improve the primary outcomes of patient fatigue interference and caregiver burden as compared to education/support. The model for each outcome will include the main effects of time (as categorical) and condition (ACT vs. education/support) and the time-by-condition interaction. Time will be dummy coded using two dummy variables, with baseline as the reference group. A treatment effect will be evidenced by a significant time-by-condition interaction. If the treatment effect is significant, follow-up tests (R emmeans) [67] will be performed to examine group differences at each follow-up.

We estimated the effect size based on the difference between study conditions on the primary outcomes of patient fatigue interference and caregiver burden at 3 months post-intervention. In our pilot RCT in advanced GI cancer, at 3 months we found a medium effect (*d* = −0.60) of condition (ACT vs. education/support) on patient fatigue interference and a small effect (*d* = −0.18) of condition on caregiver burden [21]. In contrast to our pilot, the current trial has a booster session to maintain ACT’s effects. Minimal clinically important differences (MCIDs) or clinically meaningful changes on the Fatigue Interference subscale of the FSI have been reported for cancer populations (range = 0.39–1.63) [68]. MCIDs in caregiver burden have yet to be determined. Estimates of treatment effects from pilots are imprecise [69,70]; thus, we examined results across relevant ACT trials and recommendations regarding MCIDs [71,72] to inform our power calculation. Although ACT has rarely been studied with cancer caregivers, it had a medium effect (*d* = −0.39) on dementia caregivers’ depressive symptoms – which are strongly correlated with caregiver burden [73] – relative to psycho-education at 6 months post-intervention [74]. Additionally, ACT had medium to large effects on mental health outcomes in a meta-analysis of RCTs for clinical populations [75]. In the absence of an established MCID for a self-report measure, a standardized effect size of 0.30–0.50 is recommended to estimate the MCID [71,72]. Thus, we estimate a moderate effect size of *d* = −0.40 for both primary outcomes. With *N* = 170 dyads at 3 months post-intervention (assuming 30 % attrition), we will have 85 % power (α = 0.05, two-tailed) to detect a Cohen’s *d* = −0.40 in a linear mixed model. The estimated attrition is based on our pilot data [21] and survival rates in advanced GI cancer [76].

### 3.3. Analysis for aim 2

Linear mixed effects modeling (R lme4) [66] will be used to test the hypothesis that ACT will improve secondary outcomes as compared to education/support. For outcome measures that only patients or caregivers complete, models will include main effects of time (as categorical) and condition and the time-by-condition interaction. Treatment effects will be evidenced by a significant time-by-condition interaction. For outcomes reported by patients and caregivers, multilevel modeling for dyadic data will be used [77,78]. The model will include the main effects of time, condition, and social role (patient vs. caregiver) as well as all two and three-way interactions. The time x condition x role interaction will estimate the degree to which the treatment effect is different for patients and caregivers. The *p* values from the analyses will be adjusted using the Sidak approach to control for the familywise Type I error rate due to correlated multiple secondary outcomes. If treatment effects are significant, follow-up tests (R emmeans) [67] will be performed to examine group differences at each follow-up.

### 3.4. Analysis for aim 3

Cross-lagged panel models in Mplus [79] will be used to test the hypothesis that increases in psychological flexibility will mediate ACT’s effects on primary outcomes (patient fatigue interference and caregiver burden). Outcomes will be examined in separate models that include condition, the mediator (assessed at baseline and 2 weeks post-intervention), and the outcome at all time points. To evaluate model fit, we will use the comparative fit index (CFI) and the root mean square error of approximation (RMSEA), with CFI ≥0.95 and RMSEA ≤0.05 indicating close fit [80]. The indirect effect of condition on each outcome via psychological flexibility will be quantified using the product of the two relevant effects and tested using bootstrapped confidence intervals (CIs) accounting for nonnormality [81]. A total of 10,000 bootstrapped samples will be drawn, and the indirect effect will be considered statistically significant at *p* < .05 if the 95 % CI does not include zero.

### 3.5. Analysis for aim 4

We will use the same analytic approach, as detailed above for Aim 3, to explore the extent to which changes in mindfulness/acceptance and commitment/behavior change processes mediate the effects of ACT on primary outcomes. Outcomes will be examined in separate models that include condition, both mediators (assessed at baseline and 2 weeks post-intervention), and the outcome at all time points.

### 3.6. Exploratory analyses

To inform future research, we will also explore the extent to which patient and caregiver demographics and clinical characteristics (e.g., baseline depressive symptoms, cancer treatments) moderate ACT’s effects. For these analyses, we will use linear mixed modeling (α=0.05, two-tailed). Preliminary findings or hypotheses generated from these analyses will be examined in a future trial.

## 4. Discussion

This trial addresses the top-rated QoL concern of patients with advanced GI cancer—fatigue’s interference with functioning [82]—and caregiver burden [12,36]. As fatigue interferes with patient activities, caregivers assume increased responsibilities that impact their QoL [9–11]. Evidence-based interventions for these concerns are lacking. The ACT model [22] and our pilot data [21] provide a strong rationale for this trial. In our pilot RCT, telephone-based ACT was feasible and acceptable to patients with advanced GI cancer and caregivers and showed promise in reducing patient fatigue interference and caregiver burden [21]. The current study is the first large-scale trial of ACT with cancer patient-caregiver dyads. A dyadic intervention may improve outcomes for both patients and caregivers as they reinforce each other’s practice of ACT skills. By leveraging the familial relationship, our intervention aims to improve interdependent QoL and behavioral outcomes.

ACT is well-suited to reducing fatigue interference and caregiver burden, as it emphasizes acceptance of ongoing experiences (mindfulness) and pursuing activities aligned with personal values [20]. The use of inclusion criteria for patient fatigue interference and caregiver burden allows us to target those most in need of intervention—this is rarely done in the behavioral oncology literature [83]. Another key feature of this trial is that it examines theory-driven mediators of ACT’s impact. According to the ACT model, greater psychological flexibility is the primary mediator of ACT’s effects on health outcomes [22]. The core aspects of psychological flexibility are mindfulness/acceptance processes and commitment/behavior change processes. If increased psychological flexibility mediates ACT’s effects on patient fatigue interference and caregiver burden, then this result would be consistent with the ACT model [22]. Furthermore, changes in the core aspects of psychological flexibility will be explored as mediators of ACT’s effects on outcomes. If our analysis suggests that one aspect is driving ACT’s effects, then the intervention could be refined in future trials to emphasize this mechanism.

Demonstrating ACT’s efficacy will support its adoption in cancer care, thereby addressing a critical gap in evidence-based interventions in advanced cancer. This trial will also inform further examination of ACT with dyads coping with other serious illnesses.

## Figures and Tables

**Table 1:** Summary of core components of interventions.

Acceptance and Commitment Therapy	Education/Support

• Discuss current efforts to control fatigue (if patient) or difficult thoughts and feelings (if caregiver) and their effect on quality of life • Practice mindfulness exercises with the therapist during sessions and at home (e. g., awareness of one’s breath, body scan, leaves on a stream) • Practice cognitive defusion—noticing physical sensations, thoughts, and feelings without getting caught up in them (e.g., passengers on the bus metaphor) • Observe difficult internal experiences (e. g., fatigue, thoughts, feelings) in a detached manner. This fosters a transcendent sense of self from which to compassionately observe changing experiences • Identify important values (e.g., being a loving partner, staying connected with friends) • Set realistic goals to engage in value-based actions	• Orient to the hospital and treatment team; provide overview of patient and caregiver quality-of-life concerns and discuss patient physical quality of life and symptoms • Discuss cancer-related social challenges (e.g., talking with children about cancer, employment issues); tips on managing household tasks when ill; referrals to resources for addressing social challenges • Discuss common emotional responses to cancer, including stress, anxiety, and depression, and cancer-related cognitive concerns; referrals to available mental health services • Provide overview of common financial challenges related to cancer care; referrals to resources for addressing these challenges • Discuss strategies for assessing health information on the Internet and other modalities • Review prior session topics and describe key websites with cancer information

**Table 2:** Measures and timing of data collection.

Domain	Measure	Baseline	2-weeks post-intervention	3-months post-intervention

*Primary outcomes*:				
Fatigue interference	Fatigue interference subscale of FSI (P)	✓	✓	✓
Caregiver burden	Short-form of Zarit Burden Interview (C)	✓	✓	✓
*Secondary outcomes*:				
Sleep interference	PROMIS short-form sleep-related impairment measure (P)	✓	✓	✓
Engagement in daily activities	PROMIS short-form measure of ability to participate in social roles and activities (P, C)	✓	✓	✓
Quality of life	PROMIS short-form measure of global health (P, C)	✓	✓	✓
*Hypothesized mediator*:				
Psychological flexibility	Acceptance and Action Questionnaire-II (P, C)	✓	✓	✓
*Exploratory mediators*:				
Mindfulness/acceptance	Cognitive and Affective Mindfulness Scale-Revised (P, C)	✓	✓	✓
Commitment/behavior change	Values Progress subscale of Valuing Questionnaire (P, C)	✓	✓	✓
*Demographic and medical factors:*				
Sociodemographics	Sociodemographics (P, C)	✓		
Medical comorbidity	Checklist of 8 or 9 conditions (P, C)	✓		
Functional status	Self-reported ECOG (P)	✓	✓	✓
Cancer information (e.g., date of diagnosis, cancer treatments)	Chart review	✓	✓	✓
Physical and mental healthcare use	Healthcare use interview (P, C)	✓	✓	✓
Medications	Medication interview (P, C)	✓	✓	✓
*Severity of symptoms*:				
Fatigue severity, sleep disturbance, cognitive concerns, anxiety, depressive symptoms, incontinence, and diarrhea	Fatigue severity items from FSI; PROMIS short-form measures of sleep disturbance, cognitive concerns, anxiety, and depression (P, C). PROMIS short-form measures of incontinence and diarrhea (P)	✓	✓	✓

*Note*. Administered to Patients (P) or Caregivers (C). FSI = Fatigue Symptom Inventory; PROMIS = Patient-Reported Outcomes Measurement Information System; ECOG = Eastern Cooperative Oncology Group.

## Data Availability

No data was used for the research described in the article.
